# Widening the divide: the impact of school closures on primary science learning

**DOI:** 10.1007/s43545-021-00122-9

**Published:** 2021-05-06

**Authors:** Cherry Canovan, Naomi Fallon

**Affiliations:** University of Central Lancashire, CB106 Chandler Building, Preston, PR1 2HE UK

**Keywords:** Primary education, Science education, Science capital, Widening participation

## Abstract

Prolonged Covid-19-related school closures in the UK raised concerns that science teaching and learning at primary level would be negatively impacted. This paper reports the findings of phase 1 of a study that the authors are conducting with teachers and parents to explore this issue. We found that a significant proportion of teachers were providing less science during lockdown than in the normal school week. Teachers, particularly those working in more deprived areas, reported that translating the science curriculum for home learning had been difficult, with concerns around resources, internet access and parental ability to help. Some areas of the curriculum posed particular difficulties, leading to a narrowing of topics being taught. Both teachers and parents felt that schools prioritised English and maths above science. Meanwhile some parents reported that their children had engaged in sophisticated extracurricular activities, bolstered by resources available at home and knowledgeable adult help, but others said that their children had done no science at all. Parents who had studied science at post-compulsory level were much more comfortable in helping their children with science home learning. These factors combine to create conditions which may exacerbate existing inequalities as to who can access science education and careers.

## Introduction

On 18th March 2020, faced with the threat of the Covid-19 pandemic, the UK government announced that schools across the country would close to all but the children of key workers^1^ and those deemed to be ‘vulnerable’.^2^ The closures mirrored the response of countries across the world, with UNESCO stating that ‘Most governments around the world have temporarily closed educational institutions in an attempt to contain the spread of the COVID-19 pandemic… affecting over 90% of the world’s student population,’ (“Covid-19 response,” n.d.)

The UK government had initially expected that up to 20% of children (Turner 2020) would continue to attend school; however the numbers actually attending were much lower. Government data showed that in the period 23rd March-22nd May 2020, median attendance in England was under 2%, a pattern that was mirrored in Scotland, Wales and Northern Ireland. The vast majority of the UK’s young people were, therefore, plunged into a world of online teaching supported by parents, many of whom had themselves been catapulted into a world of home working.

Although the closures were strongly supported by the UK population (“All schools are to be closed to most pupils from Friday as a result of the coronavirus. Do you think this is the right or wrong decision?” 2020), and there was considerable public resistance to the gradual reopening which began on 1st June 2020 (“Would you support or oppose all primary school children in England spending at least a month back at school before the summer holidays?” 2020), there was also disquiet from academics, charities and NGOs about the potential impacts on children of this unusual period. These ranged from the life-threatening—for example, UNICEF warned of malnutrition among the global poor caused by missing school meals (“Futures of 370 million children in jeopardy as school closures deprive them of school meals—UNICEF and WFP,” 2020)—through to serious societal harms such as increased risk and lack of visibility of child abuse (“Social isolation and the risk of child abuse during and after the coronavirus pandemic,” 2020), and more insidious long-term effects such as reduced educational opportunity magnifying existing inequalities (Burgess and Sievertsen 2020). The Sutton Trust produced a study based on research conducted in the early weeks of lockdown in the UK which showed that the impact of the pandemic on education had the potential to negatively impact social mobility (Cullinane and Montacute 2020). Regular school summer closures are known to lead to learning loss, particularly among families of low socioeconomic status (SES) (Shinwell and Defeyter 2017; Stewart et al. 2018), and it seems reasonable to suggest that longer unplanned closures might magnify such effects.

In later months, more evidence of the educational impacts of closures has emerged; the Education Endowment Foundation, for example, found that Key Stage 1 pupils’ (age 5–7) attainment in reading and maths was significantly lower, and disadvantage gaps higher, than in 2019 (Rose et al. 2021). Meanwhile, using data from the Netherlands, Engzell et al. found that “[Learning] losses are up to 60% larger among students from less-educated homes, confirming worries about the uneven toll of the pandemic on children and families” (Engzell et al. 2021).

Our study investigates the potential for one specific harm that may arise from the move to online learning—the exacerbation of inequalities in access to science, particularly among younger children. Research from Belgium (Maldonado and De Witte 2020) shows that in science, as with most other subjects, standardised test scores for primary pupils dropped significantly from 2019 to 2020. Although the Belgian authors do not examine science scores from an inequality perspective, existing research suggests that the move to home learning could amplify the difference in experiences and learning among those with the greatest and least ‘science capital’. We report on the results of surveys done with parents of primary-age children and primary school teachers in the UK during the first full lockdown period in spring–summer 2020, and make recommendations for how the science education community can work to mitigate these effects.

## Background

The average person working in science in the UK is disproportionately likely to be white, male and from an affluent background (Archer and DeWitt 2017). This matters for reasons of equity—science careers, which are often interesting, prestigious and highly-paid, should not be restricted to privileged sections of society—but also for the more practical reason that in order to meet the government’s stated aim of training more scientists in order to boost growth in the SciTech sector,^3^ we will need to enlarge the current recruiting pool for STEM degrees.

Evidence suggests that efforts to persuade young people from non-traditional backgrounds that science is ‘for them’ need to begin at an early age. The ASPIRES project, which conducted 19,000 surveys with young people aged between 10 and 14, found that science-related aspirations were steady across this age group—in other words, attitudes to science were already formed by the end of primary school. The authors state that ‘Efforts to broaden students’ aspirations, particularly in relation to STEM, need to begin at primary school.’ (Archer et al. 2013)

The effectiveness of primary science education in the UK^4^ has long been a subject for debate. In its report *Successful Science: An evaluation of science education in England 2007*–*2010*, Ofsted noted that whilst secondary-level science education had improved over the study period, ‘…there are areas that need further improvement, especially in primary schools’ (Ofsted 2011). Meanwhile the Wellcome Trust’s 2014 report *Improving primary science* noted that ‘…very few schools have access to high levels of science expertise… strategic leadership for the subject is weak’ (Wellcome 2014).

More recently, concerns have continued to be voiced about the importance placed on science in the primary curriculum, particularly in contrast with the other ‘core’ subjects,^5^ maths and English. HM Chief Inspector of Education, Amanda Spielman, said that a focus on maths and English was ‘squeezing the science curriculum out’ (Spielman 2018). Meanwhile Ofsted research found that, after science was downgraded in the primary testing regime,

Science has clearly been downgraded in some primary schools… This is likely to have a serious impact on the depth and breadth of science understanding and knowledge that pupils take with them into secondary school, which may in turn stifle pupils’ later curiosity and interest in the sciences.

Another area of concern is primary teachers’ understanding of, and confidence in, teaching science, a topic that has been discussed for decades (e.g. Harlen and Holroyd 1997; Murphy et al. 2007). A recent ‘State of the nation’ study by the Wellcome Trust (Leonardi 2019) is reasonably positive but suggests that there is still work to be done in this area:

Those responsible for science delivery are broadly confident in their ability to teach science and describe science in a positive manner… However, a significant minority are concerned that they are unable to answer pupils’ questions about science or lack confidence in undertaking formative and summative assessments.

The picture which emerges from the research of recent years, then, is one of science as a subject which, although labelled ‘core’, is in fact given significantly less prominence in primary teaching than English or maths. In addition, at the very least a significant minority of teachers lack confidence in their science knowledge or assessment capability. This in turn raises concerns that, in the sudden move to online teaching, science may have been neglected or the emphasis placed elsewhere.

This is a potentially serious issue because, for young people from backgrounds with little experience of science, school may be their only route via which to ‘level up’ with their high-science-capital peers. Science capital is a concept used to describe the web of influences and supports that some people access in order to see pursuing science as a realistic and achievable aim for their future. Studies have found (Archer et al. 2015) that high science capital has a significant relationship to science-related aspirations and post-compulsory choices. Science capital is somewhat related to social class, in that those with low science capital are more likely to be from less advantaged families. Affluence is not, however, a sufficient condition for high science capital; many other social and cultural factors contribute, and in fact the above study only classified 5% of its cohort as being in the ‘high science capital’ group.

Analysis of the factors that comprise science capital shows that family influences, such as parental interest in science, and informal activities that can happen in the home, such as reading or chatting about science, are both influential (DeWitt et al. 2016). The importance of parents in fostering an inclination towards science is well documented. For example, a study in Canada (Trache and Andres 2008) found that ‘…there is a noticeable correspondence between students with university-educated parents and the completion of science courses in high school’. The potency of certain family milieux where science futures are concerned is well described by Archer et al. (Archer et al. 2012):

We found that where middle-class family habitus, capital, and a child’s identification with science were in alignment in favor of science, the result was particularly powerful, with families able to foster and capitalize on their child’s interest, enabling them to occupy a strong and privileged position from which to potentially pursue these aspirations further. Science appeared as a ‘‘natural’’ choice within such families—albeit one that is actively nurtured and resourced.

Further discussion on the influence of parents and families on science participation can be found in Venville et al. (2013), Sonnert (2009) and Archer and Tomei (2013).

However, parents are not the only source of influence and school can also play a part; Maltese and Tai (2010), who talked to graduate students and scientists about what sparked their interest in the field, found that ‘nearly 40% of the responses from the participants indicated that school-based factors played a key role in sparking their initial interest in science’, and advise that ‘teachers must work to foster this interest so that it is not lost as students mature’.

Here we again see the potential for differential impacts of the Covid-related school closures on young people’s perceptions and learning of science. If school is reduced or removed as an influencing factor on science aspirations, children from high-science-capital backgrounds may be able to continue on a positive science trajectory thanks to parental interest and involvement which is denied to their peers from less science-focused families.

It is possible to envisage a worrying intersection between two effects identified in this review that may arise due to school closures: schools and teachers failing to prioritise science, or having difficulty in providing suitable work, in the rush to develop home learning materials, while children from different backgrounds have widely different experiences of parent-generated lockdown science activities. In order to explore the extent, if any, to which this effect occurred in practice, we decided to institute a study to address the following questions:

1. Did the move to home learning differentially affected schools’ provision of science? Was science provision impacted to a different or greater extent than that for other subjects?
2. Did some families participate in more lockdown science activities, whether school-provided or home-generated, and what are the characteristics of these families?

## Methods

In order to investigate these issues, we instigated a study to be conducted in two phases; phase 1, with data collected during the first full school closure period, and phase 2, to be conducted after schools return to normal teaching. This paper looks at the findings of phase 1. The study employs mixed methods, collecting both quantitative and qualitative data.

Phase 1 of the study consisted of two surveys, one of UK primary teachers and one of UK parents/guardians^6^ of primary-age children. When designing these surveys, our immediate concern was to avoid placing extra pressure on participants in what was a difficult and stressful situation. We therefore took a conscious decision that the surveys would be short—a maximum of 10 min to complete—and that we would rely solely on passive recruitment methods via social media to attract respondents. Both of these decisions come with drawbacks; a short survey cannot hope to cover the detail that a more lengthy instrument would, while the recruitment method inevitably meant that our sample was self-selecting and that certain groups, for example those not engaged with social media or without ready internet access, are excluded. We acknowledge these drawbacks, but felt that our responsibility to avoid causing participants stress or distress overrode them. We also took the decision that our phase 1 surveys should be anonymous, both to allow people to feel able to speak freely, and to reduce feelings of stress related to participation. Again, this approach had the drawback that it would have been possible for individuals outside of our research parameters, for example those resident outside the UK, to participate; again, we feel that under the circumstances, this choice of approach was merited.

It is also useful to note that ability to complete computer-based surveys is dependent on easy access to the relevant devices. Research has shown (e.g. Andrew et al. 2020) that children’s access to IT during the first school closure period—and by extension, that of their parents—was strongly associated with income. It is therefore likely that, due to inequalities in IT access among parental respondents, our computer-based study understates the extent of socioeconomic gaps in school experience.

Our teacher survey was restricted to current teachers in UK primary schools. It asked which of the four nations the respondent taught in and whether their school’s intake was in an area of high, moderate or low deprivation. The survey asked about the balance of subjects being taught through home learning, and how this compared to the normal school week; it also asked about the teacher’s experience of translating the usual science curriculum for the remote learning setting.

Our parent survey was restricted to UK-based parents of primary-age children. It asked what educational content families were receiving from their child’s school and how they were engaging with it, as well as what extra-curricular science activities the parents and children were pursuing. We gathered postcodes and highest level of science qualification held by the participant to act as basic measures of socioeconomic status and parental science capital.

The surveys were open for just over 3 weeks, from 29th April 2020 when ethical approval for the study was granted, to 22nd May 2020. This end date was chosen because the following week was half term for many pupils, and a phased return of some year groups to school settings began on 1st June 2020. Quantitative results were analysed using Microsoft Excel, while qualitative results were analysed using an inductive thematic process; the material was read several times to ensure familiarity, and then tagged with descriptive codes. These were then examined to develop overarching themes, and the initial codes revisited with these in mind.

After data collection and analysis for this study was complete, the UK entered a further closure period. We intend to conduct further work on the impacts of closures on primary science throughout 2021, largely once some level of normality has returned to the UK education system. This will include a series of interviews in order to look more deeply into the effects of the closures on science education, as well as a survey to measure teachers’ perceptions of comparative learning loss across subjects. However, there is value in a contemporaneous picture of the impacts of this sudden change to primary education, both in capturing a unique moment and in avoiding recall effects which are likely to be present in later studies.

## Results

### Teacher survey

In total there were 182 responses to our survey of teachers: 91 from England, 88 from Scotland and 3 from Wales. No Northern Ireland-based teachers completed the survey, and Scottish teachers were particularly responsive to our survey and were over-represented compared to the relative populations of the UK’s four nations. Of those who answered, 21% of teachers stated that their school catchments were in areas of high deprivation, 34% in moderate deprivation and 44% in areas of low deprivation. We received 10 responses from teachers at independent schools, with the remainder from state schools.

We asked teachers which subjects were covered by the work being provided to pupils who were learning at home, with options of (a) mainly maths and English; (b) most subjects but in different proportions than in normal school and c) most subjects, in roughly the same proportions as in normal school. Teachers were able to choose more than one category. In total, 36% stated that work set was mainly maths and English, while 46% stated that most subjects were being covered but in a different proportion to that in normal school. These figures indicate that, in the main, the balance of subjects being set by primary schools for home learning during the closure period differed from that in the normal school week.

When asked to give more information, many teachers stated that maths and English were being covered daily. Some noticed an increased focus on health & wellbeing, particularly in Scotland where this is a more central element of the curriculum. Other than this, a variety of approaches were described: for example, following the normal timetable; setting maths, English and one other topic per day; giving a weekly choice of activities; or covering subjects fortnightly instead of weekly.

We then asked teachers how often pupils were being asked to engage with science-related activities. Here the most common answer (119/182) was once or twice a week, with eight respondents saying that pupils were asked to do science daily. However, 30% of teachers (55/182) stated that pupils were either asked to engage with science less than once a week, or not at all.

A significant proportion of teachers (35%) stated that pupils were given less science than in a normal week, with 60% choosing ‘about the same’ and nine respondents saying that pupils were doing more science than usual. Of those who stated that their school catchment was in an area of low deprivation, 29% said that pupils were having less science engagement than normal; the equivalent figure for teachers with high-deprivation cohorts was 44%. Here we begin to see some indications that different communities may be differentially affected by the science home learning experience.

### Adapting the curriculum

Teachers were asked about the experience of translating the science curriculum to the home environment. Although a minority of teachers described the experience as unproblematic, or ‘easy’, the dominant depiction was one of difficulty. In fact of 165 responses, a total of 59 used words such as difficult/difficulty, tricky, tough, hard and even impossible to describe this experience.

In order to explore this issue further, we analysed and categorised teachers’ responses on this point as either positive, neutral, mixed or negative. Here are some typical examples of responses:

- Positive: ‘It has been a fine transition as used lots of online resources to support.’
- Neutral: ‘Reinforcement of areas covered. Practical activities and experiments with carers.’
- Mixed: ‘Useful links available and support from science centre and websites. Having the resources is the challenge at home.’
- Negative: ‘Very difficult. Children will not necessarily have the resources at home to do the same sort of practical science they would have been doing in school.’

**Fig. 1:**
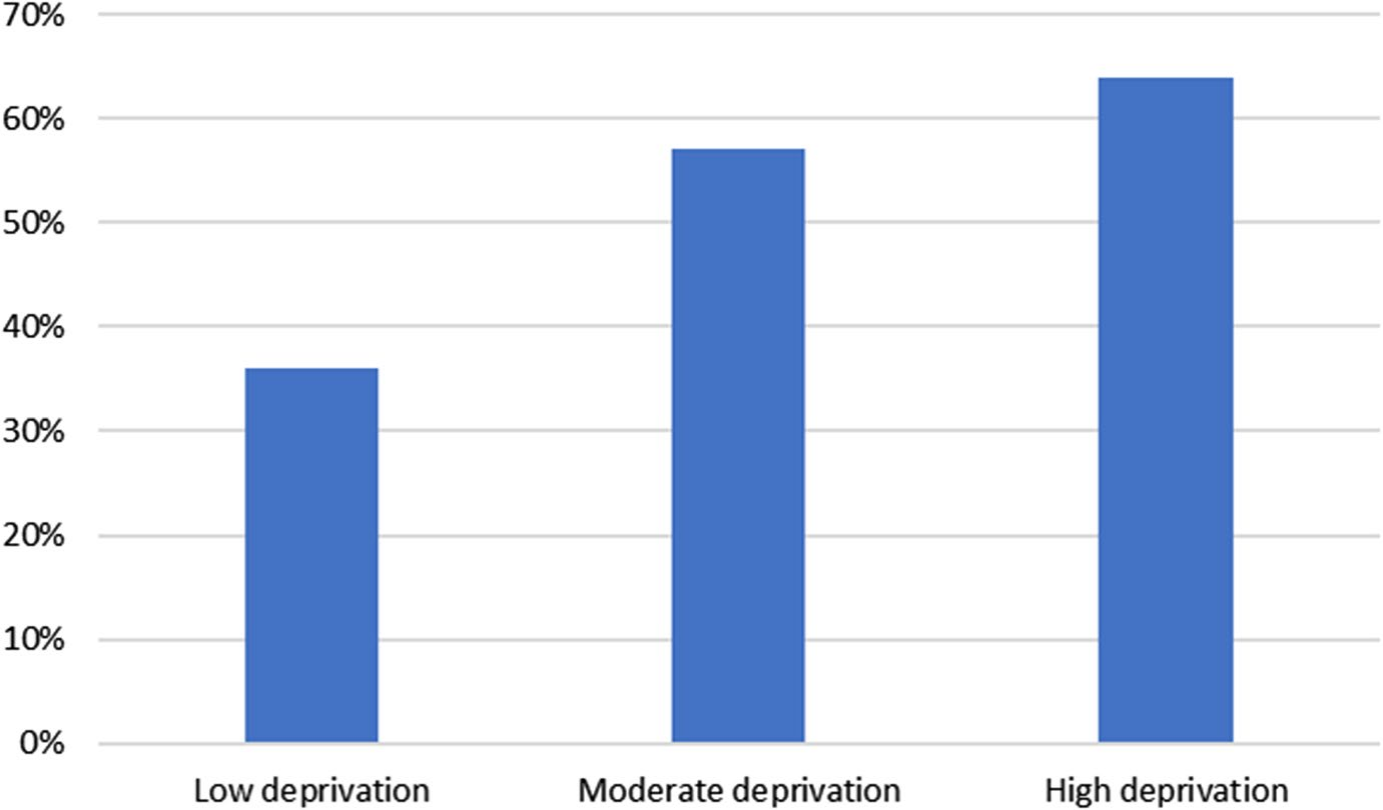
% of teachers reporting negative experiences of translating the science curriculum to the home environment, by deprivation of school catchment

This coding throws the differences between catchment deprivations into stark relief (Fig. 1). Of those who answered this question, 36% of low-deprivation-area teacher responses were coded as negative. This figure rises to 57% among teachers in areas of moderate deprivation, and 64%—nearly two-thirds—among teachers whose catchments are in high-deprivation areas. Both of these figures show a statistically-significant difference from the low-deprivation group (*p* < 0.05, Fisher’s Exact Test).

Responses from teachers in the high-deprivation group include:

It’s difficult as to conduct some experiments you need resources that parents may not have access too. In our school many do not have access to a laptop/computer only internet via the phone or smart tv.

Difficult when pupils have little prior knowledge or access to the internet. Also for some areas it is difficult to ensure that misconceptions are not reinforced by parents.

Across the board, resource availability and accessibility was the primary factor in the ease or difficulty faced by teachers. The most common difficulty discussed by teachers was in how to set work or activities that were accessible for all families irrespective of their circumstances. Teachers expressed a reluctance to place expectations on parents to provide a substantial home-learning experience at a time when they may be under significant financial pressure, juggling home working or looking after children of different ages.

Furthermore, parents’ varying level of understanding or science education and confidence to guide their children through activities was seen as more of a limitation for science learning than in the other core subject maths and English.

Ensuring knowledge is available to apply during independent activities is tricky. We cannot rely on parents having the subject knowledge themselves. Experiments are restricting for some families due to lack of resources therefore these have to be carefully considered as well.

### Comparison with maths and English

Teachers were asked how their experience with the science curriculum compared with that of the other core subjects, maths and English. Their responses suggest that for many schools and teachers there were lower expectations of science learning at home, with maths and English seen as the priority. Science was seen as posing different challenges to maths and English; while these subjects can largely be completed through written work and reading, science is necessarily more varied, involving practical elements and therefore more likely to be impacted by a lack of resources at home and parental knowledge.

As a result, science teaching was largely less frequent and less formal, and with fewer requirements for submitted work. However, a few teachers identified positive consequences to the approach, contending that science offered a more engaging and attractive prospect for both pupils and their parents:

Much less structured and more open ended. But in terms of what pupils are uploading it’s looking like the pupils are enjoy it more than literacy and maths learning.

These responses were more prevalent among teachers with low deprivation cohorts, possibly because these pupils had greater resources and parental support at home.

### Parent survey

In total, 360 parents completed our survey, with the vast majority, 329, from England. We used a simple proxy for family science capital—whether or not the parent completing the survey had participated in post-compulsory science education, i.e. A level or above. Of those who answered this question (357/360), 4% had no science qualification at all, 51% had science to GCSE, 14% had studied science at A level, 20% had a science degree and 10% had a science postgraduate degree. As OECD figures^7^ show that around 5% of adults in the UK hold a STEM degree and around 3% a postgraduate STEM degree, this is a significant over-representation of these groups. As noted in the Methods section, this type of self-selection of respondents is an unavoidable consequence of the chosen low-impact methodology.

We also analysed respondents’ postcodes to get a measure of deprivation via the national governments’ indices of multiple deprivation statistics. These statistics must be treated with caution; due to different income and employment distributions, they are not readily comparable across the four nations of the UK, and are more or less granular in terms of the geographical areas which they cover (Abel et al. 2016). In addition, 56 respondents—16% of the total cohort—did not give enough information for us to assign an IMD decile to them. With these caveats in place, the distribution for England (by far the largest participant group) was significantly skewed towards less deprived respondents, with 22% coming from IMD d1-3 areas and 41% from IMD d8-10 areas. Again, this type of self-selection bias is an inevitable consequence of the recruitment protocol.

Most respondents had children at state primary schools, with only eight specifying that their child attended an independent school. All primary age groups were well represented, with 122 parents having children in more than one year. Where this was the case, participants were asked to reply to subsequent questions with their oldest primary child in mind.

Many families surveyed were facing the challenge of co-ordinating home education while themselves working, whether at home or in the workplace. Only 148 respondents (41%) stated that an adult was available all day to engage in activities with children, while 151 said that an adult was available for some of the day and 54 that all adults were working full time. Due to the factors noted above we are reluctant to make comparisons between demographic groups, but it is striking that families living in areas of high deprivation and with lower levels of science education were much more likely to state that an adult was available all day to engage in activities with children, possibly because these parents were either not working or were in jobs that could not be done from home.

### School work

We asked parents what home learning subjects they had been set by school, and whether they had engaged with them. There was some confusion among respondents about this question, with some stating that they had engaged with school subjects which had not been set; parent and child-initiated activities were covered in a later section. However the responses give a good idea of which areas of the curriculum children engaged with during the school closure period.

By far the most commonly set subjects were maths and English, cited by 349/352 (99%) parents who answered. A much smaller proportion reported being set work in other areas—the next most common subject was art/music, selected by 69%, followed by science on 67% and history/geography on 59%. The least common subject area reported to be set by schools was foreign languages, at 26%.

Of the 356 parents who told us which activities their child had engaged with, again by far the most frequently cited were maths and English. The frequencies for all subjects can be found in Table 1. Science, as a core subject, seems to have been set more frequently than the foundation subjects (the terminology used in England for other curriculum subjects such as history and PE). However as can be seen, there is a greater proportional disparity between the ‘set’ and ‘engaged with’ figures for science than for most other subjects.

**Table 1:** Proportion of parents who reported that their child had been set, and had engaged with, school work on different curriculum subjects

Subject	Subject was set by school (*n* = 352) (%)	My child engaged with school work on this subject (*n* = 356) (%)
Maths	99	98
English	99	97
Art/music	69	63
Science	67	58
History/geography	59	55
PE	53	52
Design & tech/computing	40	38
Foreign languages	26	21

### Subject confidence

We asked how comfortable parents were helping their children with their learning overall; 155 (43%) said they were ‘very comfortable’ and 175 ‘fairly comfortable’, with only 27 stating they were ‘not comfortable at all’. There were no significant differences on this measure by eldest child’s year group or parental level of science education.

**Fig. 2:**
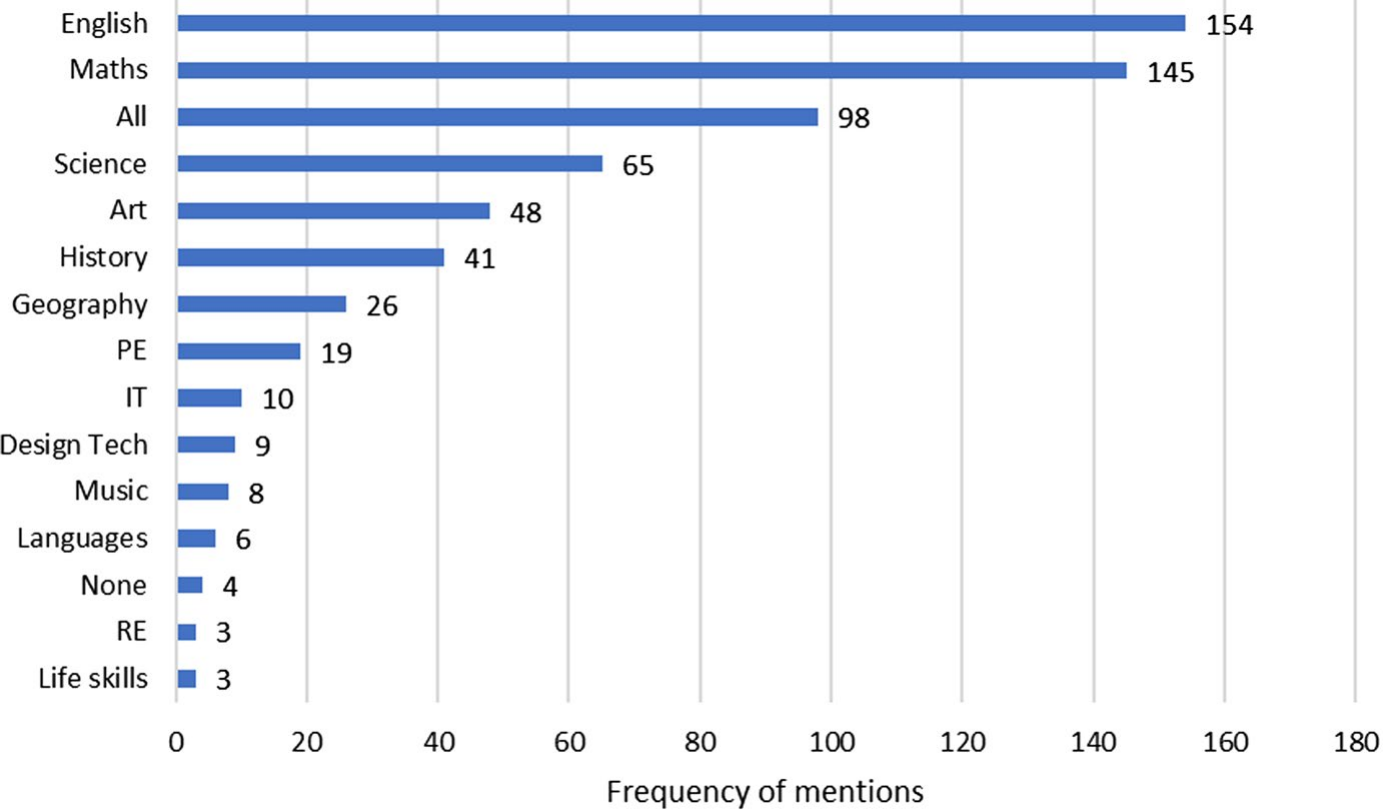
Frequency of mentions of each subject by parents when asked which subjects they felt most comfortable helping their children with

We then asked which subjects parents felt most confident helping their children with (Fig. 2). This was an unprompted, free text question. Of the 342 parents who answered this question, 29% (98) parents responded that they felt comfortable with ‘all’ of the subjects—of those, 22 had some experience in teaching professions. Maths and English were the most commonly mentioned individual subjects with 154 and 145 mentions respectively. Science was third, but much lower at 65 (just under one in five respondents). It is important to remember that our sample is biased towards individuals with higher than average levels of science education.

### National curriculum topics studied

We asked parents to give examples of science activities that children had participated in that had been suggested by the school. There were 247 responses in total, of which 37 responded that they were given no science related activities by the school (no/none/NA). Of the 211 remaining responses, the majority included references to subject-specific activities and/or the nature of the work given. Where relevant, the responses given were categorised according to the science curriculum topics set out for England by the UK government,^8^ with an acknowledgement that there may be differences in the specific topics set out by the devolved nations. The majority of parents cited one or two activities; a total of 269 subject mentions were coded.

Of necessity, our categorisation is somewhat inexact; for example, it might be hard to determine whether a parent-described activity related to ‘animals’ or ‘living things and habitats’. What is abundantly clear is that topics that fit under the broad heading of ‘the natural world’, by which we mean plants, animals, living things and habitats, were by far the most common school topics that children participated in, with a total of 127 making up nearly half of all responses. Examples of activities cited include growing seeds—the single most described activity mentioned by parents throughout all of the topics, mentioned 26 times in total—as well as habitat exploration (bug hunts, types of trees on walks) and studying the human body.

Because these three topics are taught across multiple year groups, we would expect to see them mentioned very frequently; even so, ‘natural world’ themes dominated the discourse, and this finding tied in with teachers’ reports of which subjects lent themselves to home learning.

Activities relating to materials and matter were the next most frequently cited by respondents, mentioned on 61 occasions; these were often discussed in relation to heating/cooling water or cooking processes. Another fairly common topic was forces and magnets (36), with activities including making a paper raft or boat to see if they float or sink, or making a paper plane in the examination of gravity and air resistance.

The other topics (light, earth & space, electricity, rocks, seasons, sounds, evolution and inheritance) were each cited by less than 10% of respondents. The wide differentials between topics add further substance to teachers’ comments that some lend themselves more favourably to home-learning activities.

### Extracurricular science

As well as asking about school science work, we also asked parents about science activity that had been initiated independently or from within the family. A total of 184 individuals said that their child had engaged with science-related resources or activities other than those suggested or provided by school, including BBC Bitesize lessons, other science websites and books/magazines. We also asked about joint activity, with the question ‘Have you and your child engaged together with science-related learning outside of the curriculum, such as discussing science ideas, conducting experiments or attending online events?’ to which 180 answered yes. There was significant overlap between these groups, with 144 respondents answering ‘yes’ to both questions.

It is notable that families whose children had engaged in school science were much more likely to also engage in extra-curricular science. Where a child had not done school science, just under half had engaged in other resources and/or activity with parents. This proportion rose to 70% where the child had taken part in school science activity. In total 20% of parents reported that their child had engaged with no science, either school or extra-curricular, which is concerning given that our sample was expected to be more science-oriented than average.

Common forms of extracurricular science activity included doing simple experiments, talking about science during other activities such as gardening or baking, or using online content such as BBC or YouTube videos. Of the 193 who described extracurricular activities that they had participated in, 50 respondents volunteered information that clearly involved a specific financial commitment, such as providing science kits, magazine subscriptions, books or paid-for online activity. Practical activity was popular; 53 families reported doing ‘experiments’.

Interestingly, 37 respondents gave answers that suggested they have very high science capital; these include availability of adult scientists for discussion, evidence of large amount of scientific activity or skill, and use of technical terminology. As Archer et al. (Archer et al. 2015) classified around 5% of the students they surveyed as in the high science capital group, this gives weight to the suggestion that our sample is skewed towards this group. Examples of these responses include:

Grandma and Grandpa have been delivering science demonstrations via Zoom! We have briefly looked at chromatography, making an electrical current through potatoes and we made a volcano using vinegar and bicarbonate.

We extracted DNA from an onion (wanted to show the children what DNA was).

Although we saw some suggestion that children in families where the parent had post-compulsory science qualifications were more likely to engage in extra-curricular science activities, these effects were small and will be investigated in more detail in phase 2 of the research. However what is undisputable is that these parents were, as might be expected, more confident in helping their children with science work. A total of 131/355 respondents (37%) said they were ‘very comfortable’ helping their child with science—fewer than the 43% who stated they were very comfortable helping with learning in general. However, when we split the group into those with post-compulsory science education and those without, the difference is stark. Half of the post-compulsory group were ‘very comfortable’ helping with science learning, but only 26% of the compulsory-only group placed themselves in this category. This difference did not appear for learning in general.

### Extracurricular topics

Much of the learning covered in extracurricular activity was similar to that being set by teachers—a strong focus on nature, for example minibeasts, habitats, life cycles and the human body. Sometimes this was enhanced by equipment available in the home, for example ‘We have cut up a flower and looked closely at it under microscope.’ The focus on materials and matter seen in school work, as represented by discussions of boiling/freezing water or changes during the baking process, was also represented.

Outside of the natural/domestic world, a few ‘standard’ activities appeared numerous times—‘volcano’ making (e.g. ‘Mentos and Coke’), lava lamp making and growing crystals, possibly because instructions and/or kits for these are widely available. Whether or not the science behind these activities was being accurately communicated remains an open question.

Some families went beyond these ‘standard’ science activities to explore different areas. A common focus was on space, a topic often popular with primary-age children. One parent commented:

We’ve watched the Disney Plus documentaries about space and following the space centre’s Facebook live feeds. We’ve also watched some of the kids’ storybook readings from the International Space Station. He likes space…

Astronaut webinars, the NASA website and ‘looking at the stars’ were also featured. Some practical activities were also mentioned, for example looking at the planets through a telescope or making a model Solar System.

Other, less commonly mentioned activities concerned pH and indicators, electronics and circuits, and light, including reflection, refraction and rainbows. Despite wide variation, most if not all curriculum topics were being covered by at least some parents at home.

### Parents’ views on science home learning

We asked parents to comment generally on science learning during the school closure period, and 85 did so. Of these, 38 comments were about science that had been provided by the school, and the vast majority of these were more or less critical. Although a few parents were understanding about schools’ situations—‘I think schools are doing the best with what they have’—many more were disappointed with the amount and/or quality of schools’ science offerings:

Science seems to be the lowest priority from his school (and the one he’s most disappointed about as it’s his favourite!)

Some respondents, while disappointed, felt that they understood the pressures that led to a decision to deprioritise science:

Science experiments are often resource-led which can be difficult. Teachers won’t know what type of equipment families have available to them.

Science seems to have been given far less focus than English or maths which is understandable as primary schools are measured and judged on their English and Maths results.

Another group of parents (23 responses) voiced concerns that were specifically related to their own ability to co-ordinate science learning. Sometimes this was due to a lack of resources:

Practical experiments better at engaging kids but time consuming and not all resources were readily available in lockdown.

For other parents, however, the limiting factor was their own knowledge and/or confidence.

I think my child would enjoy doing something science related bit I just don’t know where to start.

Finally, a handful of parents commented that they were prioritising science because it was more fun or interesting for their children than other subjects:

We are doing more science related work than anything else but that is because my two boys are keen on science over anything else so easy to engage them in these activities.

## Analysis and discussion

### Has the move to home learning differentially affected schools’ provision of science?

It is clear that the answer to the above question is yes. More than a third of teachers (36%) stated that the work they were sending home for pupils was mainly maths and English, and almost that number (30%) said that science was set less than once a week or not at all. A significant minority (35%) said that science was set less often than in the normal school timetable.

Teachers reported the difficulties faced in translating the science curriculum for use in the home, with concerns about lack of resources in the home, lack of internet access, and whether parents had the knowledge to support the activity and correct misconceptions all present. Some teachers explicitly saw this as an equity issue, and were unwilling to set activities that only part of the class would be able to engage in. By contrast, most found maths and English more straightforward to set, as they involved mainly bookwork, and many suggested that these were also a greater priority for their school. Parents were also more comfortable in supporting these subjects.

Science was not only restricted in terms of time, but also in the range of what was taught. When we coded parents’ descriptions of the work their children had participated in, nearly half of all responses referred to areas of the curriculum broadly characterised as ‘the natural world’. Materials & matter and forces & magnets were also mentioned frequently, but all other curriculum topics—light, Earth & space, electricity, rocks, seasons, sounds and evolution—were mentioned by less than 10% of parents. This chimed with teachers’ comments that some topics were easier to adapt for home learning. Although we would expect to see nature/biology topics mentioned frequently, as they are taught across multiple year groups, the relative absence of some of the physical science topics is striking, and we hope to explore this further in teacher interviews in future phases of this study.

Particularly worrying were potential inequities arising from the difficulties faced by teachers in setting science. Teachers working in areas of low deprivation were much more likely to be positive about translating the science curriculum to home learning than their counterparts in areas of higher deprivation. By contrast, nearly two-thirds of teachers working in areas of high deprivation had found the experience negative; they were also more likely to state that less science than normal was being taught than their counterparts in higher-SES areas. From our results, this disparity seems to be the result of worries about resources, internet access and family’s ability to help. We have not seen evidence that this perception of difficulty is due to lack of science confidence on the part of teachers, although we will return to this question in phase 2 of the study.

These findings suggest a potential widening of differences in science learning amongst primary aged pupils with those from more deprived backgrounds, already at a disadvantage in normal educational conditions, put at an even greater disadvantage during this period of educational interruption.

### Are some families participating in more lockdown science activities, and what are the characteristics of these families?

It is clearly the case that some families are participating more in science learning during the school closure period. While nearly all parents/guardians reported that their children had engaged in school maths and English, this figure fell to 58% where science was concerned—although it is worth noting that this is higher than for most of the ‘foundation’ subjects. It is notable that there was a greater disparity between the amount of work set for science and the amount actually engaged with than for most other subjects.

Many parents noted that their child had accessed science resources other than those provided by school, and/or that they had participated in science activities with their child. However 135 families—around 38% of the cohort—had done neither. These families were much more likely not to have participated in school science; under half of families who had not done school science did extracurricular activities, while the figure was 70% for those who had engaged in school work.

Although much extracurricular activity was focused in the same areas as the school work being set, particularly in relation to the natural world and the relationship of science to domestic activity, parents overall reported a broad range of subjects of study; space was particularly popular, but our parental reports covered the whole curriculum. Many parents were able to supplement such learning with extra resources or technology, either previously owned or bought especially for the period; these included telescopes, microscopes, science kits, books and magazines. Others reported being able to call on science-skilled friends or family for conversations, activity ideas or even personalised online lessons. For both parents and teachers there was a minor theme around the purpose of science lessons during school closures as an opportunity for a fun activity, something that could be done as a family.

In these findings, we begin to see the emergence of distinct groups of science ‘haves and have-nots’—some families engage in both school-provided and extracurricular science learning, in some cases supported by a wealth of resources and/or input from other adults knowledgeable about science. Other families do none of these things; in fact 20% of the sample had done no science whatsoever. It is not necessarily the case that science-oriented families did more related activities than they would normally; rather, that they had the know-how and interest to continue with science in a situation where other families did not know where to start, and/or were not receiving input from school.

However what the characteristics of such families are is to some extent an open question. Theory would suggest that families from areas of higher-SES and where parents hold post-compulsory science qualifications would engage disproportionately in science learning; however this effect, while seen, is somewhat weak. We postulate that this may be due to two effects: family situations during the lockdown period, and the nature of our sample. There was more adult availability in lower-SES households and those without post-compulsory science education; this may be a mitigating factor against this potential source of inequality. We must also note that our sample was self-selecting; the decision to complete a survey about primary learning may indicate an interest in the subject that works against other effects. We hope to tease out these effects in the next phase of the study.

It is worth noting that parental science confidence was one area which was indubitably affected by post-compulsory learning; those with science A levels and above were twice as likely to describe themselves as ‘very comfortable’ helping their children with science than those with compulsory science only. As one area of concern for teachers was how well parents could support science learning and whether they could accurately correct misconceptions, this is an area where those with high science capital are likely to have an edge.

## Conclusions

We would summarise our findings thus:

1. In a significant minority of schools, less science was taught during the school closure period than would be taught in a normal week. The curriculum taught during the period was narrower than would normally be the case, focused on the natural world and materials/matter, with a number of subject areas (e.g. light, rocks, evolution) reported by less than 10% of parents. Many teachers and parents felt that their school prioritised the teaching of maths and English above science.
2. Teachers described a great deal of difficulty in translating the science curriculum to home learning. Concerns about moving the curriculum to the home setting included a lack of resources in the home, difficulties in accessing the internet, and concerns about parents’ ability to support science learning.
3. These difficulties and concerns were reported more frequently by teachers working in areas of higher deprivation, suggesting that families in these areas are likely to be disproportionately impacted by changes in school science provision.
4. Parents reported that their children engaged in school-provided science learning to a far lesser degree than for maths and English. Around half of families had engaged in extracurricular science learning or activity, and this was much more prevalent among those who had also taken part in school science. One-fifth had done no science at all, school or extracurricular.
5. Although many families had engaged with similar nature-based topics to the school work, a wide range was present including a particular focus on space. Some families supported science learning through resources such as kits and other equipment and by conversation with knowledgeable adults. Parents with post-compulsory science education were much more confident in helping their children with science, although not with education overall.

The above findings suggest a potentially concerning impact on both science learning overall and in exacerbating existing inequalities. The breadth and quantity of school science has been diminished, opportunities for participation as standard have disappeared, and the expertise of the teacher has been removed. Nevertheless, some families—the science ‘haves’—have continued to engage with the schoolwork provided, as well as facilitating extracurricular experiences enhanced by use of family resources and networks. Meanwhile the science ‘have nots’ may have seen less work set by school, due to teacher concerns about internet access, resources and parental support. These families may have struggled to find extracurricular activities to participate in, to understand the science that lay behind them, or to provide the necessary materials. Children in a significant minority of these families will have done no science at all during the closure period.

We know that science attitudes and motivations are set at a young age, so the gap that looks likely to have been widened by the effect of the closures must be addressed swiftly in order to reverse negative impacts on efforts to widen participation in science. A good place to start would be a temporary relaxation of National Curriculum rules to allow schools to focus on science areas that were difficult to teach at home (e.g. light, rocks). As schools seem to have taken quite widely varying approaches to teaching science at home, a unifying plan to catch up on science at local authority level would also seem warranted. In addition, non-school science enrichment providers and widening participation organisations should focus efforts on curriculum areas that were underprovided during the period; funding bodies should consider whether emergency grants could be made to schools in areas of high deprivation specifically to provide science enrichment activities.

The UK—and indeed the rest of the world—will be dealing with the continuing impacts of school closures and other Covid-19-related societal changes for much time to come. However, in this one area, we now have concrete evidence that the conditions are fertile for pre-existing gaps in access to science education and careers to widen. Action taken now could prevent a lasting detriment to our widening participation efforts.

## Data Availability

The datasets used and/or analysed during the current study are available from the corresponding author on reasonable request.

## Footnotes

1 eg medical staff, social workers, supermarket staff https://www.gov.uk/government/publications/coronavirus-covid-19-maintaining-educational-provision.

2 Those with a social worker or entitled to special needs support https://www.gov.uk/government/news/schools-colleges-and-early-years-settings-to-close.

3 https://www.gov.uk/government/topical-events/the-uks-industrial-strategy.

4 Education in the UK is a devolved matter, and primary curricula differ between England, Scotland, Wales and Northern Ireland. In terms of science, broadly speaking, England and Wales specify science as a stand-alone subject, while Scotland and NI teach on a cross-curricular basis.

5 In England, the three ‘core’ subjects are maths, English and science. The situation varies across the four nations; in Scotland, the core subjects are literacy, numeracy, and health and wellbeing.

6 Henceforth referred to as ‘parents’.

7 https://stats.oecd.org/.

8 https://www.gov.uk/government/collections/national-curriculum.
